# Multichannel ballistocardiography: A comparative analysis of heartbeat detection across different body locations

**DOI:** 10.1371/journal.pone.0306074

**Published:** 2024-08-01

**Authors:** Martina Ladrova, Filip Barvik, Jindrich Brablik, Rene Jaros, Radek Martinek

**Affiliations:** Department of Cybernetics and Biomedical Engineering, Faculty of Electrical Engineering and Computer Science, VSB–Technical University of Ostrava, Ostrava, Czechia; Helwan University Faculty of Engineering, EGYPT

## Abstract

The paper presents a validation of novel multichannel ballistocardiography (BCG) measuring system, enabling heartbeat detection from information about movements during myocardial contraction and dilatation of arteries due to blood expulsion. The proposed methology includes novel sensory system and signal processing procedure based on Wavelet transform and Hilbert transform. Because there are no existing recommendations for BCG sensor placement, the study focuses on investigation of BCG signal quality measured from eight different locations within the subject’s body. The analysis of BCG signals is primarily based on heart rate (HR) calculation, for which a J-wave detection based on decision-making processes was used. Evaluation of the proposed system was made by comparing with electrocardiography (ECG) as a gold standard, when the averaged signal from all sensors reached HR detection sensitivity higher than 95% and two sensors showed a significant difference from ECG measurement.

## Introduction

Heart activity monitoring using non-electrical principles mainly for telemedicine applications [1] is becoming popular due to many advantages. It can be measured non-invasively using ballistocardiography (BCG) that is applicable in different environments, such as in whole-day or work routine (wearable devices) [2], within vehicles (chair-based devices) [3, 4], or sleep and healthcare environments (bed-based systems) [5] including magnetic resonance imaging (MRI) [6, 7]. Thus, one can see that the implementability into many types of unobtrusive sensor designs (beds, chairs, etc.) is one of the main advantages of the BCG monitoring. However, it brings many other benefits, such as resistance to electromagnetic interference, low price, ability to sense both cardiac and respiratory activity, and ease of use that contributes to lower patient’s stress and increased workflow of operating medical staff [1, 8].

**Fig 1:**
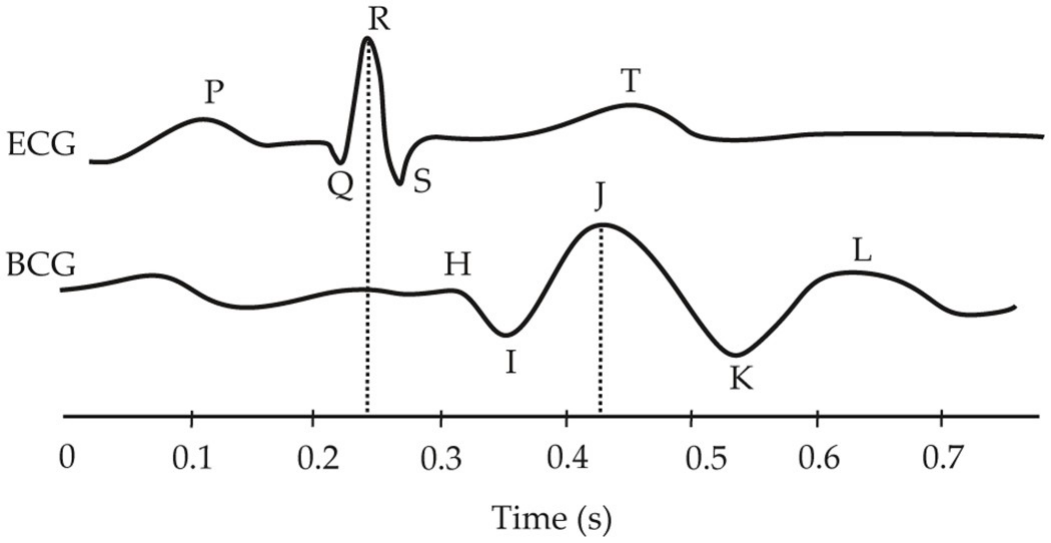
Example BCG signal and its delay versus ECG.

The BCG signal represents vibrations caused by expulsion of blood from the heart and its subsequent flow through the bloodstream. Within one heart period, three parts of BCG signal can be differentiated (see Fig 1):

- presystolic—rarely seen F and G waves,
- systolic—H, I, J (the most dominant one), and K waves,
- diastolic—waves L—O, when L wave should be dominant in healthy subjects [1].

It is known that the usual BCG signal is delayed 100–300 ms compared to ECG record [9] and signal frequency is ranged from 0.1–25 Hz [1]. Therefore, it is worth to mention disadvantages of BCG signal measuring that are:

- susceptibility to artifacts arising from slow motion or vibrations from patient and environment,
- possible distortion of diagnostic information due to varying delay of the signal.

The purpose of this study is to propose an effective alternative approach for heartbeat detection and analysis of heart rate (HR) using BCG based on pneumatic principle. The multichannel measuring BCG system was designed and performance of HR detection was evaluated in comparison with standard electrocardiography (ECG) measurement. The study focuses on issue of sensor placement within the patient’s body and investigates the possibilities of whole-body signal estimation from multiple sensors, which could increase HR detection performance.

Validation of the proposed system aims to investigate its suitability for using in many actual applications, where the different locations of sensor placement are needed. These include HR monitoring of workers in industry environment within simultaneously developping Industry 5.0, drivers or passengers during both ground or air transport, or finally, patients both in home and hospital care, including Intensive Care Unit. A special area of using the proposed BCG monitoring system is within MRI, when the BCG signal is used not only for monitoring of patient’s health status, but also for synchronization of MR data acquisition. The previous prototypes designed for this purpose were presented earlier [10, 11].

The paper is organized as follows: Section State-of-the-Art provides state-of-the-art of BCG signal measuring and processing, Section Material and Methods describes methods used in this study, and Section Results presents the obtained results that are discussed in Section Discussion together with possibilities of further research.

## State-of-the-Art

The sensors used for BCG sensing are usually placed under the patient’s body and can be based on electromechanical [9, 12, 13], piezoelectric [14–17], accelerometry [18, 19], fiber-optic [20], hydraulic [15], or pneumatic [11] principles. Depending on the chosen type of sensor or hardware available, different signal processing methods are used for signal conditioning and analysis [21]. In general, it is necessary to pay attention to signal filtration that eliminates the following inteferences:

- subject’s movement represented by huge unsteady changes in signal amplitude,
- respiratory activity manifesting by signal baseline modulation or H and J waves distortion,
- noise and vibrations from the environment [1, 5].

The most often way how to pre-process BCG signal is in the literature Butteworth band-pass filter (BPF) usually with the range of frequencies from 0.5 to 10 Hz, but it can vary depending on the application, see Table 1.

**Table 1:** Overview of state-of-the-art processing methods.

Study	Sensor principle	Processing method	Set parameters	Results
[22], 2021	Fiber-optic	MODWT-MRA	4th level, *Biorthogonal 3.9*	Average MAE = 4.83 ± 1.13 bpm
		CWT	*Gaussian 2*	Average MAE = 3.34 ± 1.54 bpm
		Template matching	–	Average MAE = 4.06 ± 1.61 bpm
[23], 2020	–	WT	*Daubechies 3–6*, 7th level	
		RMS filter	*N* = 200	
		Segmentation	1-minute window (7500 samples)	SE (0.895–0.968)
		Energy transform	–	PPV (0.753–0.928)
		Mean filter	30 samples	
		Machine learning (SVD)	–	
[16], 2020	Piezoelectric (18 channels)	Adaptive filter	–	Average MAE = 31 ms (27–38)
		Butterworth BPF	7th order, 2–10 Hz	
		Template correlation	10 s sliding window	
		Linear regression model	1728 samples	
			(training 80%, testing 20%)	
		Kalman filter	–	
[24], 2020	Accelerometer	WT	*Morse* wavelet	Accuracy > 95%
		Segmentation	700 ms window	
[12], 2019	Electromechanical film	BPF	0.7–10 Hz	SE = 97% PPV = 94.6% Accuracy = 94.4%
		Segmentation + normalization	30 s sections	
		Stationary WT	6th level	
		Machine learning (SVD)	Validation dataset 30%	
[25], 2019	Database signals (*PhysioBank*)	WT	3rd level, *Symlet 5*	–
[17], 2019	Piezoelectric	Butterworth BPF	0.7–10 Hz	MAE = 2.17 bpm (1.12–4.4)
		Hilbert transform	–	
		Viterbi decoding	–	
[26], 2018	Fiber-optic	Butterworth BPF	3rd order, 5–20 Hz	Relative error = 4.77%
[27], 2018	Fiber-optic	MODWT	4th level, *Biorthogonal 3.9*	MAE = 10.12 ±4.79 bpm
[15], 2016	Hydraulic	Butterworth BPF	0.4–10 Hz	Mean Error = 0.755 bpm
		Machine learning (eFUMI)	5 min training + 5 min testing	
[14], 2015	–	Cepstrum	–	Average Mean Error = 1.6%

Another often used approach is a signal filtration based on Wavelet Transform (WT) [23]. However, there is no general recommendation for the ideal WT parameters, so they are usually chosen experimentally. For example, Azhaginiyan et al. [25] proved an increase of signal quality using 3rd level of decomposition and *sym5* wavelet, Hytonen et al. [24] filtered the signal by *Morse* wavelet, Wen et al. [23] used the *Daubechies* family of wavelets, and Sadek et al. [27] found *bior3.9* wavelet as the best when using Maximal-Overlap WT (MODWT). In another study, Sadek et al. [22] compared the performance of three methods: multiresolution analysis of the MODWT (MODWT-MRA), continuous wavelet transform (CWT), and template matching. In this study, the CWT method (using *gauss2* wavelet and 4th level decomposition) achieved the best average results among the compared methods.

After the pre-processing step, HR is usually estimated by J-wave detection. This process can be managed using different methods, such as machine learning (ML) [12, 15], derivation and binarization of the signal [20], Kalman filtration [16], Hilbert transform and FFT [17], or detection of the higher energy in scalogram (using WT) [24]. In [14], heart activity was estimated indirectly, when the cepstrum is computed in the individual windows that represents time between two consecutive heart beats.

## Material and methods

The BCG measuring system was designed with the aim to cover as close as possible contact between sensors and measured tissue to reach an effective transmission of the measured signal. This section describes methodology used for BCG sensing and signal processing, including an analysis of the obtained data.

### Measurement system

**Fig 2:**
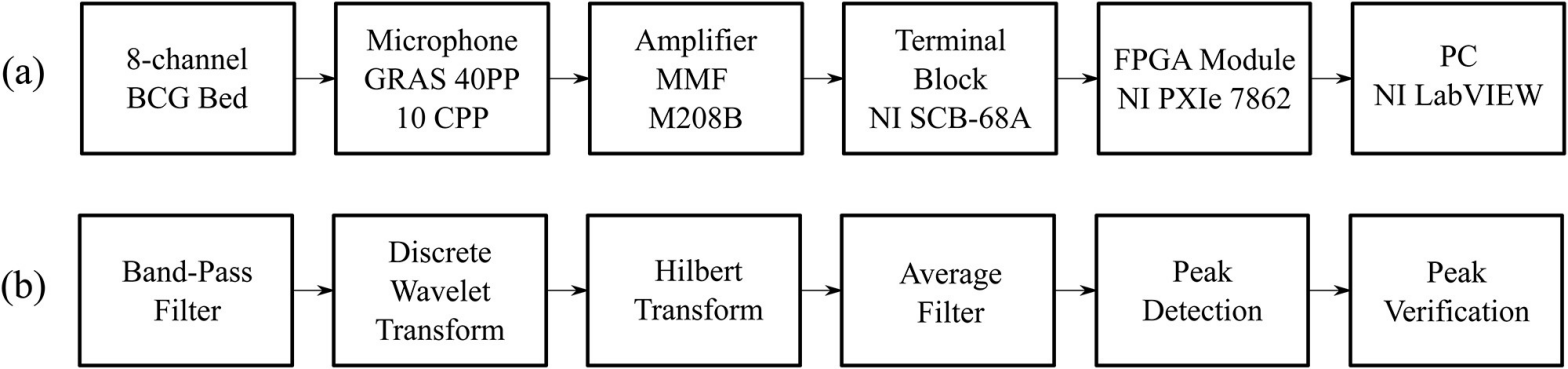
Block diagrams of (a) measurement hardware and (b) signal processing procedure.

The sensors in the form of closed pneumatic pillows were used for sensing BCG signal. These pillows are made of non-metallic materials, such as polyvinylchlorid (PVC), polyuretan (PUR), and silicone, see [10]. The hardware part of the measuring system is based on Virtual Instrumentation by National Instruments (NI). The sensors are connected to microphones GRAS 40PP-10 CCP (frequency range 10 Hz–20 kHz, resolution 50 mV/Pa) and MMF M208B amplifier (frequency range 0.1 Hz–100 kHz, voltage output ±10 V) for power supply of microphones and gain of the signal. Amplifier outputs were then interfaced to terminal block NI SCB-68A and reconfigurable Field-Programmable Gate Array (FPGA) module NI PXIe-7862 (placed in 10-slot chassi NI PXIe-1092). In Fig 2 you can se block diagrams of measurement hardware and signal processing procedure.

**Fig 3:**
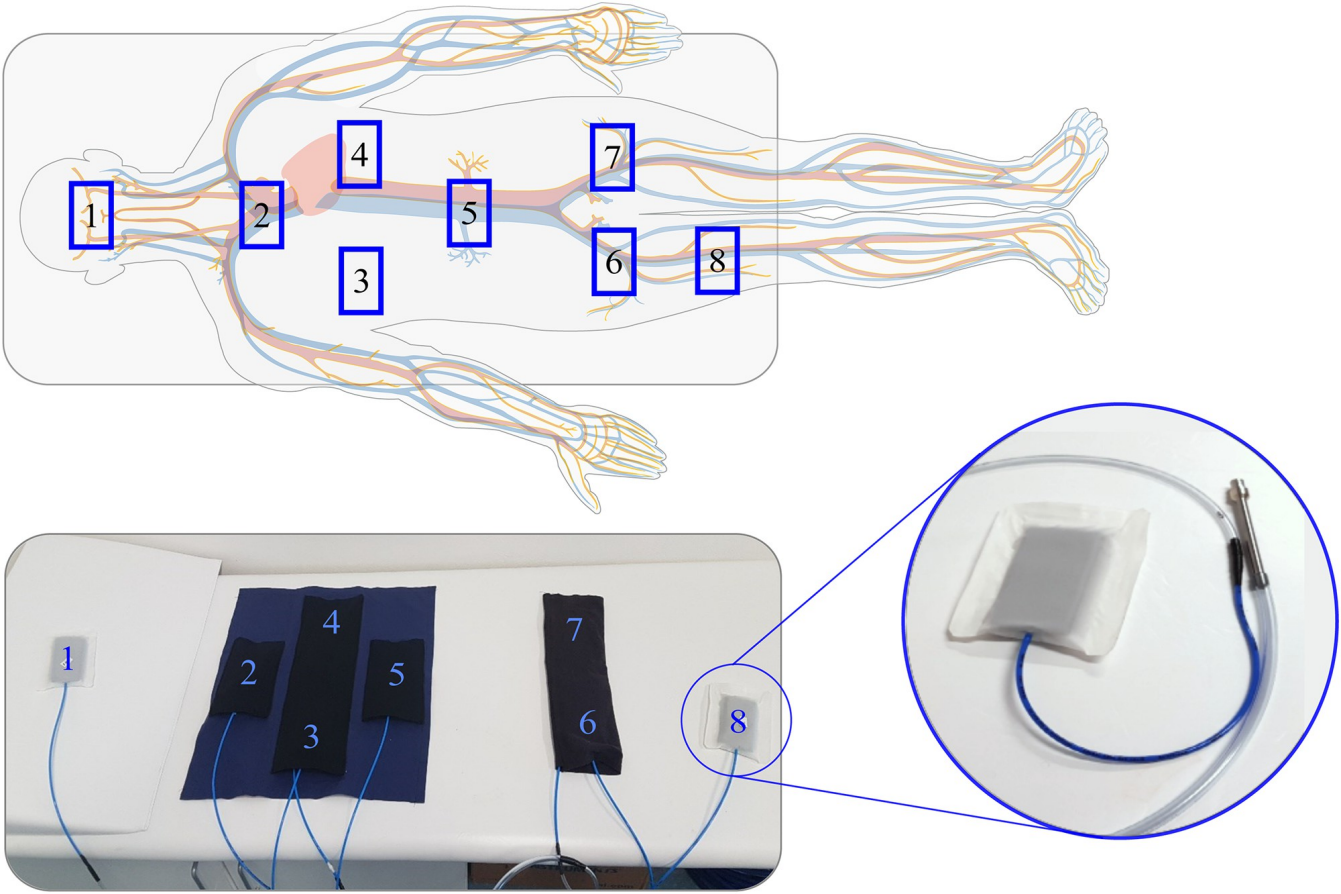
Sensor distribution during experimental measurements.

The sensors were placed in 8 locations (S1–S8) under patient’s body (in supine position), so that their layout corresponded to the distribution of large arteries in the human body and the sufficient pressure of the contact body part on the sensor was ensured (see Fig 3):

- S1—carotid artery (under the head),
- S2—arch level of aorta (under the neck),
- S3 and S4—heart level of aorta (under the chest),
- S5—renal level of aorta (under the loins),
- S6 and S7—iliac arteries (under the buttocks),
- S8—femoral artery (under the leg).

### Dataset

All experiments were performed in accordance with the relevant regulations and approved by the Ethics Committee of Technical University of Ostrava. Experiments were completely safe, using certified hardware from National Instruments. The recruitment period of participants lasted from 31/01/2022 to 17/03/2022. All of them signed an informed consent form to agree to the publication of the results, where all their data will be anonymised.

The measurement was performed on 27 health subjects (16 men and 11 women, see Table 2), each lasting about 10–15 minutes. Together with BCG signals, ECG reference was recorded to serve as a gold standard. The sampling frequency was set to 2.5 kHz. The placement of the sensors was selected in locations of large vessels under the patient’s body to compare a signal quality from different body parts. Thus, eight sensors were placed in the locations shown in Fig 3.

**Table 2:** Dataset parameters.

Parameter	Value/Mean (Range)
# of subjects	27
Age (year)	23.63 (21.00–29.00)
Height (m)	1.76 (1.58–1.94)
Weight (kg)	73.41 (50.00–99.00)
BMI (kg/m^2^)	23.33 (18.21–28.09)
Measurement time (min)	approx. 10–15
Sampling frequency (kHz)	2.5
# of channels	9 (8 BCG + ECG)

### Signal processing

**Fig 4:**
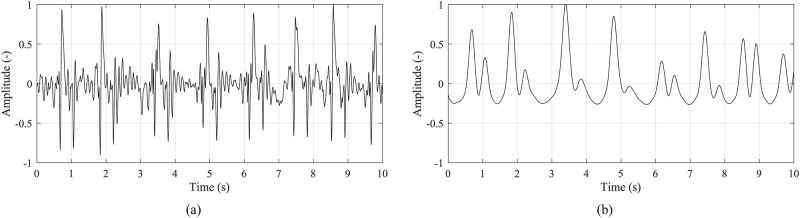
Short sample of a (a) raw measured signal and (b) signal after processing.

For precise peak detection, the pre-processing steps should be performed to obtain high-quality signal and distinguishable J-waves. The processing steps described bellow were chosen according to literature survey and performed in LabView software, National Instruments. The signals before and after the pre-processing are shown in Fig 4.

#### Band-pass filter

Butterworth BPF with cut-off frequencies of 0.5–15 Hz was used to eliminate the most of the previously mentioned artifacts. For preserving the precise time information, Zerophase filter was used, which allowed an analysis of delay of BCG (J-wave) beside ECG (R-wave). The filtered signal was further normalized, so that the amplitudes were unified for easier determination of peak detection threshold.

#### Wavelet transform

Wavelet transform, as a method advantageous for non-stationary signal conditioning [28], was used for better recognition of the desired signal components. Different types of maternal wavelets and levels of decomposition were experimentally tested based on knowledge from review of methodology: *Daubechies* (*Db4* and *Db6*), *Symlet* (*Sym4*), and *Biorthogonal* (*Bior3.5*).

#### Hilbert transform

The next step included the second power providing non-linear amplification of the signal (i.e., increasing higher amplitudes more than lower ones) and Hilbert transform for calculation of signal envelope, when the real signal is converted into complex signal [29], which improves performance of some operations of signal processing. The obtained signal was then filtered by moving average filter with window width selected as 125 ms.

#### Signal averaging

**Fig 5:**
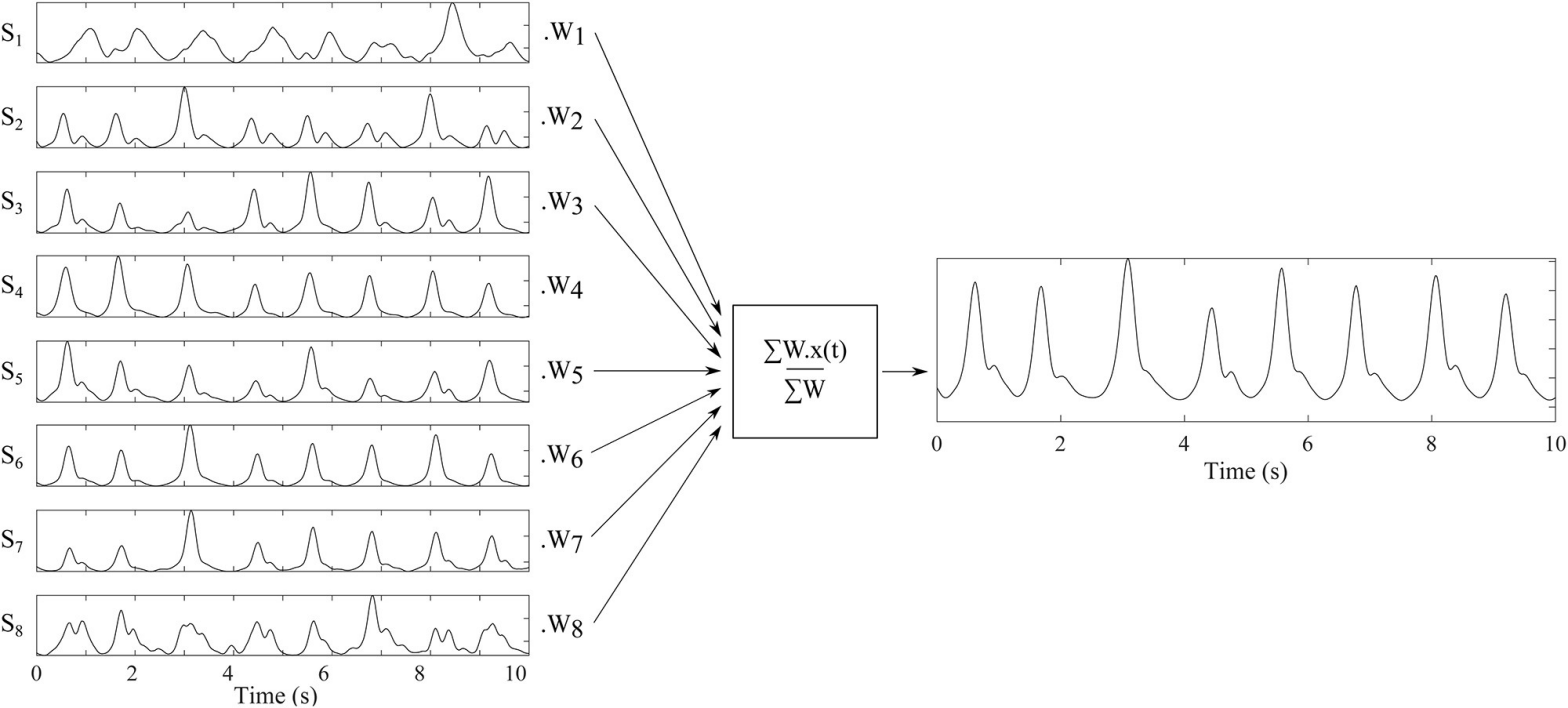
Averaging of the processed signals from S1–S8 in order to amplify peaks representing heart beats.

For reduction of motion artifacts present in the signal and improve of J-wave detection quality, the weighted average of the signal from all sensors was calculated according to Eq (1):

$$\begin{array}{c} y\left(t\right)=\frac{\sum_{i=1}^{N}W_{i}x_{i}\left(t\right)}{\sum_{i=1}^{N}W_{i}}, \end{array}$$ (1)

where the weights were chosen experimentally based on signal morphology and J-wave detection success as *W*_1_ = 0.8, *W*_2_ = *W*_3_ = *W*_4_ = 1.8, *W*_5_ = 1.0, *W*_6_ = *W*_7_ = 1.2, and *W*8 = 0.7 for each sensor S1–S8, respectively (see Fig 5).

### Peak detection

From the pre-processed signal, J-waves were determined using the Peak Detector VI (NI LabVIEW). A threshold *thr* for J-wave detection was selected statistically as:

$$\begin{array}{c} thr=\mu +0.4\sigma , \end{array}$$ (2)

where *μ* is mean of the BCG signal and *σ* is its standard deviation. Due to unbalanced peak amplitude or artifacts occuring, there are two situations that had to be controlled by the added algorithm:

- *Extra peak*—interval between two consecutive peaks shorter than 0.2 s; amplitudes of peaks were compared and the lower one removed.
- *Missing peak*—interval between two consecutive peaks longer than 1.5 s; detection was repeated with lowering threshold and detected peaks were verified according to the peak-to-peak intervals—if one of the intervals was shorter than 0.6 ⋅ *median* and the interval between the peaks being checked is shorter than 1.4 ⋅ *median*, the middle peak is removed.

From the detected peaks, HR in *beats per minute* (bpm) is calculated from the *periods* between each two consecutive heart beats (sec) as:

$$\begin{array}{c} HR=\frac{60}{period}\left(bpm\right). \end{array}$$ (3)

### Reference ECG

For evaluation of HR detection quality, one-lead reference ECG was recorded simultaneously with BCG using electrodes placed on upper limbs and left leg. The signal was pre-processed using Butterworth BPF with cut-off frequencies 5–15 Hz. Then, second power of the signal was calculated and this output was again filtered by zero-phase Butterworth low-pass filter with a frequency of 2 Hz. R-peaks were found using Peak Detector VI (NI LabView) with a window width of 3 and threshold set as half of the signal average.

### Evaluation parameters

The verified J-waves from the previous process were compared to the R-peaks detected in ECG with presumprion that J-wave should be located in the specified interval around R-peak, which was chosen as a median of delay between R-peaks and J-wave (R-J intervals), i.e, ±50 ms (expected interval). The results can be classified as:

- *True positive (TP)*—J-wave is detected within the expected interval around R peak,
- *False negative (FN)*—J-wave is not detected within the expected interval around R peak,
- *False positive (FP)*—peak is detected out of the expected interval around R peak.

These indeces were further used for calculation of sensitivity (SE) and positive predictive value (PPV):

$$\begin{array}{c} SE=\frac{TP}{TP+FN}100\left(\%\right), \end{array}$$ (4)

$$\begin{array}{c} PPV=\frac{TP}{TP+FP}100\left(\%\right). \end{array}$$ (5)

Another evaluation step included statistical comparison of HR obtained by BCG and ECG. Since the HR values can be considered as dependent pair data, Wilcoxon pair test was used after rejecting the normality of data using non-parametric test. For better interpretation of results, mean absolute error (MAE) was also calculated:

$$\begin{array}{c} MAE=\frac{1}{n}\sum_{i=1}^{N}\left|HR_{ECG}-HR_{BCG}\right|. \end{array}$$ (6)

## Results

This study has a primary goal to evaluate HR detection performance using the proposed measuring and processing system. However, results of other two subgoals are also described—investigation of WT parameters used for BCG pre-processing and time delays between BCG (J-wave) and ECG (R-peak) signals.

### Wavelet transform parameters

To enhance the peak detector performance as much as possible, different parameters of WT were tested. Their selection was based on the literature review, the visual similarity of the wavelet with the shape of the ideal signal and by experimental verification of the wavelet effect on signal decomposition. After a broader experimental testing, wavelets *Db4*, *Db6*, *Sym4*, and *Bior3.5* were chosen for a detailed comparison of results. The results in Table 3 were obtained using signals from one subject and averaged across sensor locations. The best results for enhancing the recognition of the desired signal components were achieved by *Bior3.5* wavelet and decomposition level 6. Thus, these parameters were used for further analysis. Contrary, the worst performance was revealed in *Db6* and decomposition level 5.

**Table 3:** Results of pre-processing using different wavelet parameters.

Wavelet	Level	SE			PPV		
		Mean (%)	Min (%)	Max (%)	Mean (%)	Min (%)	Max (%)
Db4	5	60.0	37.8	81.6	56.1	36.6	82.2
Db4	6	78.2	45.3	96.1	78.7	46.5	96.1
Db4	7	68.9	22.7	96.7	68.6	19.8	96.8
Db6	5	34.3	3.2	71.7	32.3	2.5	69.5
Db6	6	71.4	36.6	87.2	71.7	36.5	87.7
Db6	7	73.4	41.1	97.2	73.1	38.9	97.3
Sym4	5	58.3	39.3	79.7	55.1	36.7	80.7
Sym4	6	78.6	40.5	97.5	79.0	41.7	97.5
Sym4	7	71.8	38.5	96.3	71.6	34.4	96.5
Bior3.5	5	80.8	61.5	93.9	80.5	54.8	94.4
**Bior3.5**	**6**	**87.4**	**58.9**	**99.7**	**87.7**	**59.3**	**99.7**
Bior3.5	7	70.2	38.6	95.7	70.3	35.8	95.7

### R-J interval analysis

Before analysis of peak detection, a delay between R-peak of ECG and J-wave of BCG was investigated as a difference of time of their occurence (sec) for each sensor. The goal was to determine a degree of delay difference when measuring from distant parts of patient’s body, see Table 4. According to statistical analysis, normality of dataset was rejected (Shapiro-Wilk test, *α* = 0.05). Thus, Kruskal-Wallis test (*α* = 0.05) about agreement of medians was performed, which revealed statistically significant difference of R-J delay depending on the sensor location (*p* − *value* < 0.001). The following homogeneous groups of sensors were found using Dunn’s post-hoc analysis: (S1, S3, S5, S6, S7), (S1, S6, S7, S8), and (S2, S4, S5).

**Table 4:** Delays of BCG signal from individual sensors.

Sensor location	Median (s)	Min; Max (s)
S1	0.181	(0.063; 0.502)
S2	0.087	(0.025; 0.443)
S3	0.139	(0.086; 0.393)
S4	0.100	(0.048; 0.171)
S5	0.145	(0.106; 0.211)
S6	0.175	(0.068; 0.225)
S7	0.174	(0.068; 0.220)
S8	0.226	(0.135; 0.412)

### Peak detection performance

The obtained results of SE and PPV from 27 measurements are summarized in Table 5 for the individual sensors (S1–S8) and average signal (*S*_*AVG*_) as median, interquartile range (IQR), and extremas (minima and maxima). The most stable detection quality was achieved from *S*_*AVG*_, when SE > 95% in 20 cases and PPV > 95% in 23 cases, followed by S7, S6, and S4. The worst results were obtained from S1 and S8.

**Table 5:** Results of SE, PPV, and difference between HR obtained by BCG and ECG.

	SE			PPV			Difference of HR Medians			
Sensor location	Median (IQR) (%)	Min (%)	Max (%)	Median (IQR) (%)	Min (%)	Max (%)	Median (95% Interval) (bpm)	*p* − *value* (-)	*p* − *value* < 0.05 # of subjects (%)	MAE (bpm)
S1	**51.86** (25.54;73.18)	5.26	98.48	**55.50** (24.86;73.96)	3.97	99.53	**-0.21** (-1.48; 0.45)	0.701	16 (59.26)	10.29
S2	**84.61** (68.55;96.45)	11.28	99.62	**90.79** (72.10;97.40)	13.43	99.62	**0.20** (0.02; 0.90)	0.015	16 (59.26)	5.53
S3	**84.98** (66.28;93.37)	25.83	98.06	**89.38** (67.12;95.94)	26.81	99.67	**0.05** (-0.08; 0.29)	0.310	13 (48.15)	5.11
S4	**94.52** (87.68;98.22)	3.35	99.74	**98.21** (92.75;98.89)	2.59	99.85	**0.09** (-0.01; 0.26)	0.077	12 (44.44)	3.32
S5	**77.87** (66.35;91.53)	35.16	99.20	**81.03** (68.76;94.88)	35.45	99.46	**-0.06** (-0.31; 0.10)	0.500	14 (51.85)	5.37
S6	**94.68** (90.13;97.76)	30.86	99.56	**97.01** (91.52;98.95)	33.95	99.61	**0.03** (-0.01; 0.13)	0.110	8 (29.63)	3.15
S7	**95.74** (81.46;98.19)	44.97	99.74	**96.99** (84.99;99.12)	46.60	99.87	**0.01** (-0.02; 0.09)	0.464	6 (22.22)	4.52
S8	**54.62** (44.62;77.67)	11.07	97.40	**60.53** (46.76;78.48)	11.09	98.75	**-0.30** (-0.68; -0.11)	0.004	12 (44.44)	8.01
*S*_*AVG*_	**97.84** (94.58;98.72)	86.08	99.87	**98.75** (96.48;99.14)	88.85	100.00	**0.01** (-0.01; 0.04)	0.280	2 (7.41)	1.77

Table 5 also presents the results of statistical analysis, including point estimation (median) and 95% interval estimation of medians of differences between HR obtained by BCG and ECG, p-values obtained by Wilcoxon test, and MAE. In ideal case, both HR curves are identical, so the difference of medians is zero. According to the results, difference from zero is statistically significant (*α* = 0.05) in the case of S2 and S8. However, the highest number of subjects with the statistically significant difference was obtained by S1 and S2 (59.26% of records) and the greatest MAE values were found in S1 and S8. Differences from other sensors are not statistically significantly different from zero, when the smallest difference was achieved in average signal—no statistically significant difference was achieved in 25 records (92.59%), followed by sensor S7 (77.78% of records) that corresponds with the obtained PPV and SE values.

## Discussion

The obtained results show that the proposed system is adequate alternative to standard ECG for HR detection, when providing safe, low-cost, and easy-to-implement technology useful for many areas of application. Besides the used novel hardware sensor prototype, processing part is also developed and presented to enhance a quality of HR detection from mechanical heart manifestations—BCG signal.

As one of the early steps in BCG signal conditioning, pre-processing using WT was used since it is the suitable and very popular tool in the area of non-stationary biological signals. Althlough there are several attempts in the literature to find an optimal parameters settings for BCG signal denoising, there is no general recommendation and the set parameters differ across studies, see Table 1. In this study, wavelets from family *Daubechies*, *Symlet*, and *Biorthogonal* available in Advanced Signal Processing Toolkit (NI LabVIEW) were selected for the performance analysis. The best results were achieved when using wavelet *Bior3.5* (mean *SE* = 97.4%), but in future research, other types of maternal wavelets could be tested, e.g. using software platforms enabling a wider range of maternal wavelets types.

Evaluation of the detected peaks showed that the placement of the sensor is crucial for precise HR monitoring. The best suitability of measuring was proved by sensors placed under buttocks and chest (S4, S6, S7). These sensor positions showed proper contact of the sensor with the subject’s body due to the amount of soft tissues and localization near the heart or large vessels. Contrary, the worst results were achieved by sensors S1 and S8, located under head and thigh. This is probably caused by the weak pressure of the leg on the sensor, leakage of the soft tissues allowing sufficient pressure transmission, or large amount of movement of these distal body parts causing poor signal-to-noise ratio. Also, the precision could be affected by different positions of subjects. This issue was not investigated in this study, because of the present focus of authors on MRI applications, where the patients are forced to lie still on their back. However, it should be taken into account in case of the implementation into areas such as home monitoring.

Also, the performance of individual sensors varied across the subjects, as can be seen in the case of in general successful sensors, e.g. S4, when one subject reached the lowest values (*SE* = 3.4% a *PPV* = 2.6%), which can be caused by a poor contact between sensor and subject’s body. This situation proves an advantage of multichannel BCG, when the other sensors compensate a measurement error. This can be useful for implementation of the sensors e.g. into beds, chairs or wearable technologies, when many artifacts can arise due to subject’s movement or noise from environment. Totally highest SE and PPV values were reached by averaging all the measured signal, when *SE* = 96% and *PPV* = 97.2%. However, a disadvantage of the average signal is loss of information about delay of the signal, which can be crucial in some applications, e.g. MRI triggering.

The results showed that the signal delay significantly depends on the sensor location (it increases proportionately with a distance from heart). The post-hoc ananalysis revealed that S2 and S4 (the closest sensors to the heart) made up a homogenous group significantly differing from other sensors, except S5. However, more clear conclusion would be made if analysing a higher number of evaluated sensors.

Although an assessment of individual BCG channels revealed the most suitable positions for sensing, the signal morphology and contact with patient’s body is highly individual. Thus, an automatic approach for selection of the best-quality signals instead of manual investigation would be beneficial. The high-quality signals can be then used for signal averaging and selecting its weights or directly for heartbeat detection to improve its performance. This will be a subject of further research, which will focus on development of classification model based on machine learning or deep learning.

The significant limitation of the present study is testing of only healthy subjects around 23 years old. Further research will focus on monitoring of non-standard signals, such as pathological states of heart activity, measuring in various positions and on various subjects (age, body constitution), or recognition of patient’s movement (caughing, snoring, etc.). The profound investigation of effects of different pathologies and other signal variances on the signal morphology and thus, detectability of desired peaks, would significantly increase a precision of HR detection and allow to develop a system for early recognition of the most predictable conditions and pathological states that would be a breakthrough in home monitoring and early diagnostics.

## Supporting information

S1 File BCG signal of proband 1. (MAT)

S2 File BCG signal of proband 2. (MAT)

S3 File BCG signal of proband 3. (MAT)

S4 File BCG signal of proband 4. (MAT)

S5 File BCG signal of proband 5. (MAT)

S6 File BCG signal of proband 6. (MAT)

S7 File BCG signal of proband 7. (MAT)

S8 File BCG signal of proband 8. (MAT)

S9 File BCG signal of proband 9. (MAT)

S10 File BCG signal of proband 10. (MAT)

S11 File BCG signal of proband 11. (MAT)

S12 File BCG signal of proband 12. (MAT)

S13 File BCG signal of proband 13. (MAT)

S14 File BCG signal of proband 14. (MAT)

S15 File BCG signal of proband 15. (MAT)

S16 File BCG signal of proband 16. (MAT)

S17 File BCG signal of proband 17. (MAT)

S18 File BCG signal of proband 18. (MAT)

S19 File BCG signal of proband 19. (MAT)

S20 File BCG signal of proband 20. (MAT)

S21 File BCG signal of proband 21. (MAT)

S22 File BCG signal of proband 22. (MAT)

S23 File BCG signal of proband 23. (MAT)

S24 File BCG signal of proband 24. (MAT)

S25 File BCG signal of proband 25. (MAT)

S26 File BCG signal of proband 26. (MAT)

S27 File BCG signal of proband 27. (MAT)
